# Electrochemical sensor for determination of butylated hydroxyanisole in real samples using glassy carbon electrode modified by [Co(HL)2Cl2] nano-complex

**DOI:** 10.5599/admet.1703

**Published:** 2023-03-02

**Authors:** Mahbubeh Fazli, Niloufar Akbarzadeh-T

**Affiliations:** Department of Chemistry, University of Sistan and Baluchestan, P.O. Box 98135-674, Zahedan, Iran

**Keywords:** Schiff base ligand, cobalt(II) nano-complex, butylated hydroxyanisole, voltammetry

## Abstract

A new mononuclear Co(II) complex with the formula [Co(HL)2Cl2] (**1**) (HL= N-(2-hydroxy-1-naphthylidene)-2-methyl aniline) has been synthesized and characterized by Fourier transform infrared spectroscopy, UV–Vis, elemental analysis and single crystal X-ray structure analysis. Single crystals of the complex [Co(HL)2Cl2] (**1**) were obtained through slow evaporation of an acetonitrile solution at room temperature. The crystal structure analysis revealed that the two Schiff base ligands create a tetrahedral geometry via oxygen atoms and two chloride atoms. The nano-size of [Co(HL)2Cl2] (**2**) have been synthesized by the sonochemical process. Characterization of nanoparticles (**2**) was carried out via X-ray powder diffraction (XRD), scanning electron microscopy (SEM), UV-Vis, and FT-IR spectroscopy. The average sample size synthesized via the sonochemical method was approximately 56 nm. In this work, a simple sensor based on a glassy carbon electrode modified with [Co(HL)2Cl2] nano-complex was developed ([Co(HL)2Cl2] nano-complex/GCE) for convenient and fast electrochemical detection of butylated hydroxyanisole (BHA). The modified electrode offers considerably improved voltammetric sensitivity toward BHA compared to the bare electrode. Applying linear differential pulse voltammetry, a good linear relationship of the oxidation peak current with respect to concentrations of BHA across the range of 0.5–150 μM and a detection limit of 0.12 μM was achieved. The [Co(HL)2Cl2] nano-complex/GCE sensor was applied to the determination of BHA in real samples successfully.

## Introduction

Butylated hydroxyanisole, a substituted phenolic compound, is one of the potent chemical antioxidants used as a preservative in food, food packaging, animal feed, cosmetics, pharmaceutical preparation, rubber and petroleum products. It is also used as a vitamin A stabilizer. BHA is added to food products to improve their stability, especially to prevent rancidity in products containing lipids or fats. BHA is exclusively used in food products because of its ability to remain active even at high temperatures while cooking or baking [1-3]. Although they are powerful in protecting product quality in food distribution, excess antioxidants added to food might produce toxicities or mutagenicities and, thus, endanger people's health [4,5]. Thus, it is necessary to reliably determine the amounts of BHA in food or other products.

Many methods, including spectrophotometry [6], gas chromatography [7], high-performance liquid chromatography [8], micellar electrokinetic capillary chromatography [9] and electrochemical methods [10,11] have been developed for the determination of BHA. Electrochemical sensors with excellent ability for the determination of electroactive substances, especially food samples, have been suggested as a powerful analytical tool in recent years [12-18]. Electrochemical sensors are fast, portable and affordable. However, in many cases, the detection limit was insufficient when traces of these compounds had to be determined. The results might also be irreproducible due to surface fouling, requiring high oxidation potentials and being time-consuming [19-26]. Chemically modified electrodes (CMEs) improve the sensitivity and selectivity of electrochemical analysis by improving the kinetics of the reaction via an electrocatalytic process at the CME surface [24-35].

Recently, more attention has been focused on the synthesis and application of nanoparticles (NPs), and on properties of NPs such as high electrical conductivity, high surface area, and chemical stability [36-40]. The NPs can be used to promote electron transfer reactions when it is used as electrode material in an electrochemical device. Therefore, the modification of the electrochemical interface by nanostructures is one of the recent approaches used extensively in the development of sensing platforms [41,43].

Schiff base ligands are among the most common coordinating ligands in coordination chemistry [44], through a direct forward condensation reaction between an amine and a carbonyl compound [45]. During the past few decades, considerable attention has been paid to the chemistry of metal complexes of Schiff bases containing nitrogen and other donor atoms [46]. This may be attributed to their stability, biological activity and potential applications in many fields, such as oxidation catalysis, electrochemistry [47], and biological properties, such as antibacterial, antifungal and antioxidant activities [48,49]. In general, the antibacterial and antifungal activities of Schiff bases are attributed to the presence of lone pair of electrons on the nitrogen atom and the electron-donating character of the double bond of the azomethine group [50]. Facile and inexpensive synthesis, allied with the wide range of structural and electronic features of Schiff bases and their coordination complexes, has increased interest in chemically modifying electrodes with these compounds. Therefore, the resulting electrodes have been used as sensors and/or probes in a variety of fields. All this interest has culminated in the development of low-cost sensors. In particular, the many structural and electronic properties of Schiff bases and their transition metal nano-complexes allow them and the analyte to establish different interactions, which improves sensor selectivity and sensitivity [51-56].

In this work, Schiff base ligand [HL] and its cobalt(II) complex (**1**) with (N-(2-hydoxynaphtalen)2-methyl aniline) Schiff base ligand were synthesized. The complex was characterized using spectroscopic techniques such as FT-IR, UV-Vis and elemental analysis. The crystal structure of title complex was determined by X-ray crystallography. The Schiff base cobalt nano-complex (**2**) was prepared by the sonochemical method. This synthesis was performed without the use of any surfactants. The nano-sized complex (**2**) was identified using XRD and SEM techniques. Also, we developed a voltammetric sensor based on [Co(HL)_2_Cl_2_] nano-complex modified glassy carbon electrode for the detection of BHA. Under optimal conditions, the sensor displayed excellent sensitivity, low detection limit and rapid response toward the analyte. Moreover, this developed sensor was applied in the determination of BHA in real samples successfully

## Experimental

### Materials and instrumentation

^1^H-NMR spectrum was accomplished with Bruker AVANCE3 3-300MHz spectrometer using DMSO-d6 as solvent and at room temperature (298 k). Fourier-transform infrared spectra were performed on a PerkinElmer FT-IR spectrophotometer model spectrum two with KBr discs in the range of 4000-400 cm^-1^. For elemental analyses of new compounds, a Costech ECS 4010 CHNS Elemental Analyzer was utilized. UV-Vis spectra were recorded in the range of 200–800 nm on Optizen view 2120UVplus Spectrophotometer ver1-2. Single crystal measurements were done with Rigaku OD Supernova, equipped with Atlas S2 CCD detector and Mo-Kα radiation from a micro-focused sealed X-ray tube. The data reduction, scaling and absorption correction were made in CrysAlis PRO. MCE was used for the visualization of electron density maps.

All the electrochemical measurements were carried out on a PGSTAT302N potentiostat/galvanostat Autolab consisting of a traditional three-electrode system: a bare or modified glassy carbon electrode (GCE) as the working electrode, an Ag/AgCl as the auxiliary electrode and a Pt wire as the counter electrode. Solution pH values were determined using a 713 pH meter combined with a glass electrode (Metrohm, Switzerland). BHA and other chemicals used were analytical grade and were purchased from Sigma Aldrich and Merck. Orthophosphoric acid was used to prepare the phosphate buffer solutions (PBSs), and sodium hydroxide was used to adjust the desired pH values (pH range between 2.0 and 9.0).

### Synthesis of Schiff base ligand [HL]

The ligand (HL: N-(2-hydroxy-1-naphthylidene)-2-methyl aniline) was synthesized by adding a solution of 2-hydroxy-1-naphthaldehyde (0.17 g, 1 mmol) in 10 mL of ethanol to a solution of 2-mehyl aniline (0.11 ml, 1 mmol). Then, the mixture was refluxed for 6 h. The resulting yellow solution was placed at room temperature. After 24 h, yellow crystals of [HL] were formed. The Schiff base ligand was characterized by ^1^H-NMR, FT-IR, elemental analysis and single-crystal X-ray diffraction: mp:(154 °C);(yield: 88 %);(MW: 261.31); Anal. Calc. for [C_18_H_15_NO]: C82.73;H6.12; N 5.79; Found: C82.70; H6.09; N5.76. FTIR (KBr, cm^-1^), 3421ν (N-H), 1620 ν(C=N), 1161 ν(C-O), 1479 ν(C=C); ^1^H-NMR (300 MHz, DMSO d_6_, ppm) 9.61 (1H, s, CH=N), 16.05 (1H, OH), 2.40 (3H,CH_3_) [37].

### Synthesis of complex (1)

**Scheme1.**
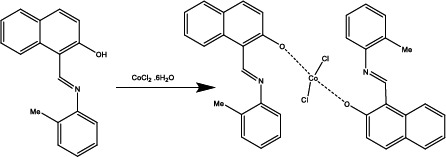
Synthetic route for complex **(1).**

The Schiff base ligand (HL: N-(2-hydroxy-1-naphthylidene)-2-methyl aniline) (0.26 g,1 mmol) was dissolved in 10 ml of methanol and to this yellow solution, a solution of [CoCl_2_.6H_2_O] (0.12 g, 0.5mmol) in 10 ml of methanol was added dropwise. The reaction mixture was refluxed for 6 h. The complex was precipitated, and the precipitate was filtered and dried at room temperature. Green crystals of [Co(HL)_2_Cl_2_] complex suitable for single crystal X-ray diffraction were obtained by the crystallization from the acetonitrile solution of the precipitate after two weeks. The complex was characterized by UV-Vis, FT- IR, elemental analysis and single-crystal X-ray diffraction (Scheme 1). mp:250 °C; (yield: 85 %); (MW: 652.5); Anal. Calc. for [C_36_H_30_Cl_2_CoN_2_O_2_]: C66.27; H4.63; N4.29; found: C66.32; H4.69; N4.27. FT-IR (KBr, cm^-1^), 1619 ν(C=N); 1146 ν(C-O), 535 ν(Co-O).

### Synthesis of nanocomplex (2)

A solution of CoCl_2_.6H_2_O (0.12 g, 0.5 mmol) in methanol (10 mL) was positioned in a high-density ultrasonic probe for 10 min. Then to this solution, 10 ml of methanolic solution of Schiff base ligand (0.26 g, 1 mmol) was added dropwise. The resultant solution was then irradiated for 60 min at 60 °C with a power of 100 W. The obtained brown precipitate was filtered and dried in air. mp: 245 °C; (yield: 87 %); (MW: 652.5); Anal. Calc. for [C_36_H_30_CoCl_2_N_2_O_2_]: C 66.27; H 4.63; N 4.29; found: C 66.34; H, 4.69; N,4.27. FT-IR (KBr, cm^-1^): 1621 ν(C=N); 1147 ν(C-O), 534ν (Co-O).

### Preparation of [Co(HL)_2_Cl_2_] nano-complex/GCE

The bare glassy carbon electrode was coated with [Co(HL)_2_Cl_2_] nano-complex according to the following simple procedure. 1 mg [Co(HL)_2_Cl_2_] nano-complex was dispersed in 1 mL aqueous solution within 50 min ultrasonication. Then, 4 μl of the prepared suspension was dropped on the surface of carbon working electrodes. It remains at room temperature until it becomes dry. The surface areas of the [Co(HL)_2_Cl_2_] nano-complex/GCE and the un-modified GCE were obtained by CV using 1 mM K_3_Fe(CN)_6_ at various scan rates. Using the Randles–Ševčik equation for [Co(HL)_2_Cl_2_] nano-complex/GCE, the electrode surface was found to be 0.109 cm^2^ which was about 3.5 times greater than un-modified GCE.

## Results and discussion

### Single crystal X-ray diffraction

Green crystals of [Co(HL)_2_Cl_2_] (**1**) were obtained by slow evaporation of an acetonitrile solution. Its structure was determined by single-crystal X-ray diffraction. The complex crystallized in the monoclinic system in space group *C*2/*c* with an asymmetric unit consisting of a metal cation in a special position, one chloride anion and one disordered HL ligand. Table 1 shows the crystallographic data for complex (**1).**

All hydrogen atoms of the strongly occupied part of ligands were discernible in difference Fourier maps and could be refined to reasonable geometry. According to common practice, H atoms bonded to C were kept in ideal positions with C–H = 0.96 Å, while positions bonded to N were refined with restrained bond length. In both cases, U_iso_(H) was set to 1.2U_eq_(C, N). All non-hydrogen atoms were refined using harmonic refinement. A disordered ligand was observed in both structures. Given the scale of disorder and numerous overlaps, we decided to use molecular refinement to model the disorders. Two models were used to allow for free rotation of the tolyl group. The occupancies of two positions were refined with the sum constrained to 1. Resulting occupancy ratios were 832(2):168(2). For further information on data collection and refinement, see Table 1.

**Figure 1.**
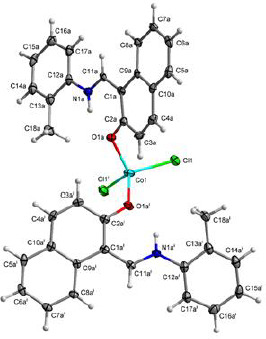
The labeled diagram of compound (1). Thermal ellipsoids are at 50% probability level symmetry code: (i) 1-x, y, 0.5-z.

**Figure 2.**
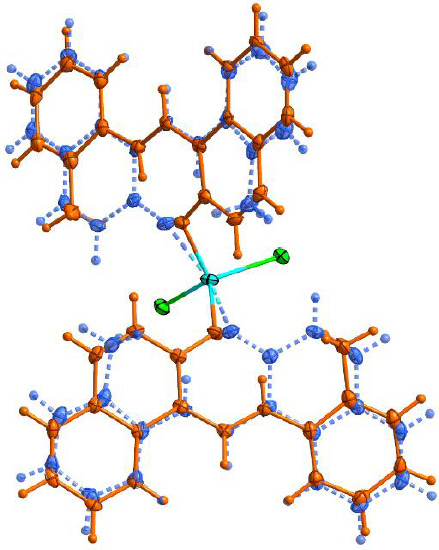
Disorder in complex (**1**), weakly occupied position depicted in blue with dashed bonds.

A view of compound (**1**) is shown in Figure 1. The occupied position is shown in Figure 2.

The cation in complex (**1**) is four-coordinate with two chloride anions and two phenolic oxygen atoms forming a slightly distorted tetrahedron. Only the oxygen atom of the neutral zwitterionic HL ligand is coordinated with the metal cation, while the presence of an azomethine proton denies the possibility of coordination via the nitrogen atom. A list of the most important bond distances and angles is reported in Table 2.

The coordination tetrahedron formed by chloride anions and the ligand with higher occupancy has only minor distortions from the ideal values with Co–Cl1 bond lengths at 2.2505(5) Å and Co–O1a bond lengths at 1.9618(16) Å. The bond angles are in the range from 104.82(6)° to 115.11(2)°. The coordination of weakly occupied oxygen atoms appears weaker compared to the oxygen atoms with higher occupancy, with Co–O1b bonding longer by 0.1602 Å compared to Co–O1a. This elongation brings the bond length closer to the length of Co–Cl1 bond, which should result in lower distortions from the ideal tetrahedron. Regardless of the similarities in the bond lengths, the distortions in the coordination tetrahedron formed with the weakly occupied ligand were even more pronounced, with the angles ranging from 99.06(19)° to 115.4(3)°.

### FT-IR spectra

**Figure 3.**
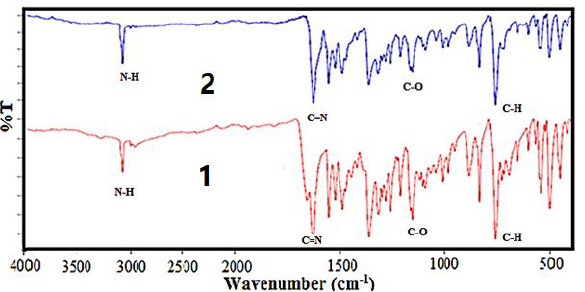
The FT-IR spectra of the complex **(1)** and its nanoparticles **(a2)**

The FT-IR analysis data is listed in Table 3. The FT-IR spectrum of the free Schiff base ligand [HL] exhibits a band in 3421 due to ν(NH^+^) and a band in 1620 cm^-1^ due to ν(C=N) azomethine. A band at 1161 cm^-1^ is assigned to the ν(C-O) phenolic group. This band has been shifted to lower frequencies for both complex (**1**) and nano-complex (**2**), in the region of 1146-1147 cm^-1^, which indicates that both compounds are formed by the coordination of the oxygen atom of [HL] to the metal ion (Figure 3)s. FT-IR spectra of both complex (**1**) and nanocomplex (**2**) show weak bands at 535 cm^-1^ and 534 cm^-1^ that assign to (Co-O), and don't show any shift due to ν(C=N) and ν(NH^+^) that this display the azomethine group of [HL] doesn't participate in complex formation [57].

### UV-Vis spectra

**Figure 4.**
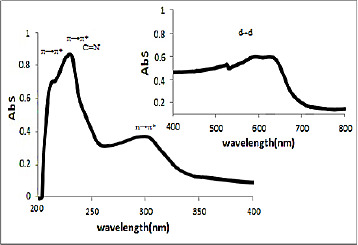
The spectrum of UV-Vis of (**1**) in methanol.

**Figure 5.**
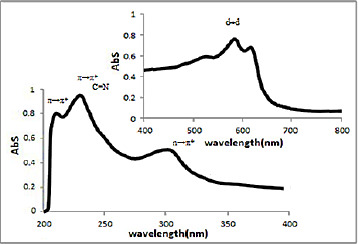
The spectrum of UV-Vis of (**2**) in methanol.

UV-Vis spectra of [HL], (**1**) and (**2**) in methanol solution contain different peaks related to the transition bands data. These absorption bands (nm) are listed in Table 2. The Schiff base ligand display two bands at 230 and 250 nm attributed to π →π* and a band at 330 nm that is assigned to n→π* transitions. In the electronic spectra of both (**1**) and (**2**), intra-ligand transitions (n→π* and π →π*) were shifted to another wavelength that this is due to the coordination of the metal to the ligands. Both of electronic spectra of complex (**1**) and nano-complex (**2**) showed three d-d absorption bands at 520-635 nm are assigned to the transition ^4^A_2_→^4^T_2_(F),^4^A_2_→^4^T_1_(F) and ^4^A_2_→^4^T_1_(P), respectively [58] (Figures 4 and 5).

### XRD and SEM

**Figure 6.**
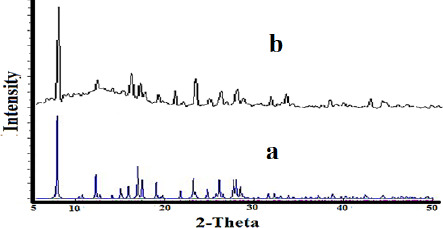
The XRD patterns of **a:** standard powder Co(II) complex (**1**) **b:** nano-sized Co(II) complex (**2**)

**Figure 7.**
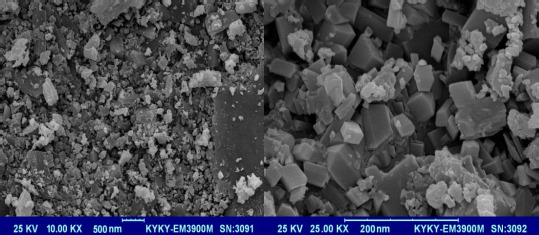
SEM images of the nanocrystal complex (**2**)

The XRD patterns of nano-sized Co(II) complex (**2**) and standard powder Co(II) complex (**1**) were obtained from single-crystal X-ray diffraction and are exhibited in Figure 6. The XRD pattern shows the crystalline phase and the nature of the complex. By investigating the location and intensity of the peaks of both patterns, it can be deduced that the diffraction angle in both complexes obtained by different methods is the same [59]. This indicates that the nano-sized complex (**2**) has a single crystalline phase that this phase is similar to that obtained by single-crystal X-ray diffraction [60]. The width of the diffraction peaks shows the nanocrystal complex (**2**) particles are of nanometer scales [61]. The particle means the size of the nanocrystal complex (**2**) was calculated using the Debye Scherrer equation. SEM images of the nanocrystal complex (**2**) are shown in Figure 7. The SEM photos also show the shape of the nanoparticles and the surface morphology of the nanocrystal complex. The average size diameter obtained from the Debye-Scherrer equation of nanocrystal complex (**2**) was approximately 56 nm.

### *Electrochemical behavior of* BHA at the surface of various electrodes

**Scheme 2.**
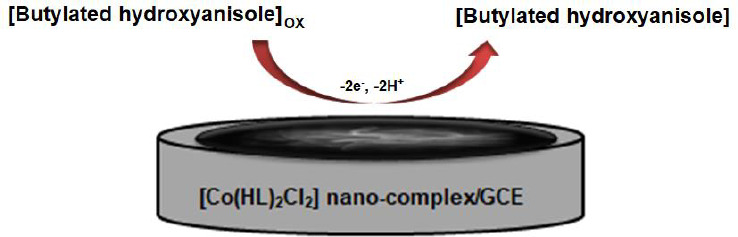
The proposed mechanism for the oxidation of BHA at the [Co(HL)_2_Cl_2_] nano-complex/GCE.

According to our knowledge, the electrooxidation of BHA is closely related to the pH value of the solution (Scheme 2). So, the effect of pH was investigated using the differential pulse voltammetry (DPV) method. The results show that the oxidation peak current increased from pH 2.0 to 7.0, and then the current conversely decreased when the pH value increased from 7.0 to 9.0. According to obtained results, pH 7.0 was chosen as the best optimal experimental condition for other experiments.

**Figure 8.**
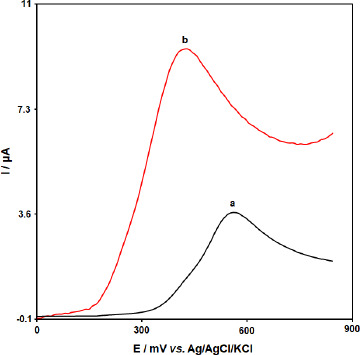
The linear sweep voltammograms of (a) bare GCE and (b) [Co(HL)_2_Cl_2_] nano-complex/GCE in 0.1 M PBS (pH 7.0) in the presence of 50.0 μM BHA at the scan rate 50 mVs^-1^.

The electrochemical behavior of BHA was investigated by linear sweep voltammetry (LSV). The linear sweep voltammograms obtained using the bare GCE and [Co(HL)_2_Cl_2_] nano-complex/GCE in 0.1 M PBS (pH 7.0) in the presence of 50.0 μM BHA are shown in Figure 8. At the bare CPE, a weak oxidation peak current (*I*_pa_ = 3.6 μA) could be seen at 0.57 V. In contrast, [Co(HL)_2_Cl_2_] nano-complex/GCE exhibited an enhanced sharp anodic peak current (*I*_pa_ =9.4 μA) at much lower overpotential *E*_p_ = 0.43 V. These results confirmed that the [Co(HL)_2_Cl_2_] nano-complex improved the sensitivity of the modified electrode by enhancing peak current and decreasing the overpotential of the oxidation of BHA.

### Effect of scan rate on the determination of BHA at [Co(HL)_2_Cl_2_] nano-complex/GCE

**Figure 9.**
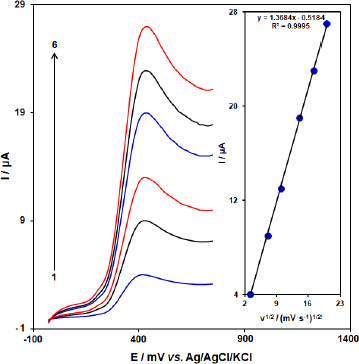
Linear sweep voltammograms of [Co(HL)_2_Cl_2_] nano-complex/GCE in 0.1 M PBS (pH 7.0) containing 50.0 μM BHA at various scan rates; 1-6 correspond to 10, 50, 100, 200, 300, and 400 mV s^-1^, respectively. Inset: variation of anodic peak current *vs.* ν^1/2^.

The influence of the scan rate (*ʋ*) on the peak currents (*I*_pa_) of BHA at [Co(HL)_2_Cl_2_] nano-complex/GCE was investigated by LSV. Figure 9 shows the voltammetric response of 50.0 μM BHA at [Co(HL)_2_Cl_2_] nano-complex/GCE at different scan rates in the range of 10 to 400 mV/s. The oxidation peak current of BHA increases linearly with increasing scan rate. A linear regression equation was obtained from the plot *I*_pa_ and *vs.* *ʋ*^1/2^ (square root of scan rate) as follows; *I*_pa_ (μA) = 1.3684 *ʋ*^1/2^ (mV/s)^2^ – 0.5184 (*R*^2^ = 0.9995) for the oxidation process, which indicates that the reaction of BHA at [Co(HL)_2_Cl_2_] nano-complex/GCE is diffusion controlled.

**Figure 10.**
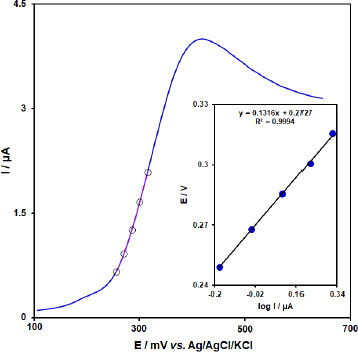
Linear sweep voltammograms response for 50.0 μM BHA with 10 mVs^-1^ scan rate. Inset: The Tafel plot derived from the rising part or the corresponding voltammogram.

In order to obtain some information on the rate-determining step, we drew a Tafel plot (Figure 10) using the data from the rising part of the current-voltage curve recorded at a low scan rate of 10 mV s^-1^ for 50.0 μM BHA. The linearity of the *E* versus log *I* plot implies the intervention of the kinetics of the electrode process. The slope of this plot can be used to estimate the number of electrons transferred in the rate-determining step. According to Figure 10 inset, the Tafel slope for the linear part of the plot was estimated to be equal to 0.1316 V. The value of the Tafel slope indicates that the one-electron transfer process is the rate-limiting step, assuming a transfer coefficient (*α*) of about 0.55.

### Chronoamperometric analysis

**Figure 11.**
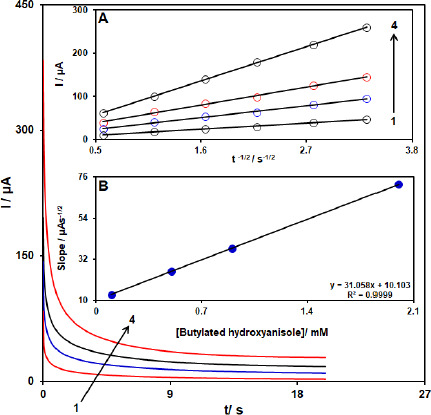
Chronoamperograms obtained at [Co(HL)_2_Cl_2_] nano-complex/GCE in 0.1 M PBS (pH 7.0) for different concentration of BHA. The 1-4 correspond to 0.1, 0.5, 0.9, and 2.0 mM of BHA. Insets: (A) Plots of *I* vs. *t*^-1/2^ obtained from chronoamperograms 1-4. (B) Plot of the slope of the straight lines against BHA concentration.

The analysis of chronoamperometry for BHA samples was performed by use of [Co(HL)_2_Cl_2_] nano-complex/GCE vs. Ag/AgCl/KCl (3.0 M) at 0.48 V. The chronoamperometric results of different concentrations of BHA in PBS (pH 7.0) are demonstrated in Figure 11. The Cottrell equation for the chronoamperometric analysis of electroactive moieties under mass transfer limited conditions is as follow:



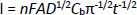



where *D* represents the diffusion coefficient (cm^2^ s^-1^), and *C*_b_ is the applied bulk concentration (mol cm^-3^). Experimental results of *I* vs. *t*^-1/2^ were plotted in Figure 11A, with the best fits for different concentrations of BHA. The resulting slopes, corresponding to straight lines in Figure 11A, were then plotted against the concentration of BHA (Figure 11B). The mean value of *D* was determined to be 8.2 × 10^-5^ cm^2^/s according to the resulting slope and Cottrell equation.

### Calibration curve

**Figure 12.**
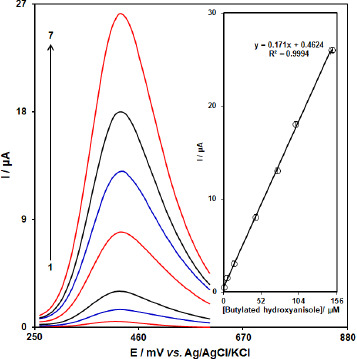
DPVs of [Co(HL)_2_Cl_2_] nano-complex/GCE in 0.1 M (pH 7.0) containing different concentrations of BHA. Numbers 1–7 correspond to 0.5, 5.0, 15.0, 45.0, 75.0, 100.0, and 150.0 μM of BHA. Inset: plot of the electrocatalytic peak current as a function of BHA concentration in the range of 0.5-150.0 μM.

Because DPV commonly has a higher sensitivity than CV, the DPV technique was applied for the quantitative detection of BHA. Figure 12 shows the differential pulse voltammograms of BHA at various concentrations using [Co(HL)_2_Cl_2_] nano-complex/GCE. As seen, the oxidation peak currents of BHA enhance gradually by increasing its concentration. The oxidation peak currents (*I*_pa_) show a good linear relationship with the concentrations of BHA ranging from 0.5 M to 150.0 μM. The linear equation is *I*_pa_/μA = 0.171C_BHA_/μM + 0.4624 (R^2^ = 0.9994) (Figure 12 (inset)). Also, the limit of detection, C_m_, of BHA was calculated using the following equation:



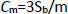



where, m is the slope of the calibration plot (0.171 μA/ μM) and S_b_ is the standard deviation of the blank response obtained from 15 replicate measurements of the blank solution. The detection limit for determination of BHA using this method of 0.12 μM was obtained.

## Conclusions

We have reported the synthesis of a Schiff base ligand and a new Co(II) complex (**1**) . The [HL]and its cobalt(II) complex (**1**) were characterized by spectroscopic techniques and elemental analysis. The single crystal X-ray diffraction analysis of the complex showed that metal ions reacted with the ligand in a 1:2 molar ratio. In the formation of the complex, oxygen atoms of two ligands are coordinated to the metal ion, and tetrahedral geometry is formed around the metal ion with two chloride ions attached to it. Also, the nanocomplex (**2**) have been synthesized by the sonochemical process. The nanocomplex (**2**) was characterized via X-ray powder diffraction (XRD) and SEM. The XRD patterns indicated both (**1**) and (**2**) compounds prepared by different synthesis methods have the same crystal structure. A sensitive and fast voltammetric method to detect the BHA based on Co(HL)_2_Cl_2_] nano-complex (**2**) modified glassy carbon electrode was established. The voltammetric investigation demonstrates that electrooxidation of BHA at the surface of [Co(HL)_2_Cl_2_] nano-complex/GCE showed very distinct characteristics due to the presence of nanoparticles of [Co(HL)_2_Cl_2_] complex on the surface of electrode. [Co(HL)_2_Cl_2_] nano-complex/GCE exhibited good catalytic activity towards the oxidation of BHA over a linear range from 0.5 to 150.0 μM with an enhanced sensitivity of 0.171 μA/μM of BHA and favourable characteristics for the determination of BHA with a detection limit of 0.12 μM. Finally the proposed method was successfully applied in the determination of BHA in real samples with satisfactory results.

## Figures and Tables

**Table 1.** Crystallographic data and refinement parameters for complex (**1**)

Compound	(1)
Formula	C_36_H_30_Cl_2_CoN_2_O_2_
Formula weight (g mol^-1^)	652.5
λ (Å)	0.71073
crystal system	monoclinic
space group	*C* 2/*c*
Hall group	-C 2yc
*a* (Å)	24.3845(7)
*b* (Å)	7.5848(3)
*c* (Å)	17.9596(9)
*β* (°)	114.763(3)
*T* (K)	95
*V* (Å^3^)	3016.2(2)
*Z*	4
*D*_calc_ (g cm^-3^)	1.437
μ (mm^-1^)	0.784
F(000)	1348
h,k,l_max_	33,10,15
θ_max_ (°)	29.34
Measured refl.	7862
Indep. refl. (*R*_int_)	3594 (0.018)
Obs. refl. (*I*>3*σ*(*I*))	3110
*R*_1_(obs)	0.0343
*wR*_2_(all)	0.0998
S	1.510
Parameters	211
*Δρ*_max_, *Δρ*_min_ (e Å^-3^)	0.35, -0.36
CCDC	1995453

**Table 2.** Selected bond lengths and angles in structures (**1**) and (**2**)

X–Y	(1) X–Y (Å)	X–Y–Z	(1) X–Y–Z (°)
Co–Cl1	2.2505(5)	Cl1–Co–Cl1^*i*^	115.11(2)
Co–Cl1^*i*^	2.2505(5)	Cl1–Co–O1a	104.82(6)
Co–O1a	1.9618(16)	Cl1–Co–O1a^*i*^	110.14(5)
Co–O1a^*i*^	1.9618(16)	Cl1^*i*^–Co–O1a	110.14(5)
Co–O1b	2.122(16)	Cl1^*i*^–Co–O1a^*i*^	104.82(6)
Co–O1b^*i*^	2.122(16)	O1a–Co–O1a^*i*^	111.97(7)
		Cl1–Co–O1b	114.6(2)
		Cl1–Co–O1b^*i*^	99.06(19)
		Cl1^*i*^–Co–O1b	99.06(19)
		Cl1^*i*^–Co–O1b^*i*^	114.6(2)
		O1b–Co–O1b^*i*^	115.4(3)

Symmetry code: (*i*) 1-*x*, *y*, 0.5-*z*.

**Table 3.** Selected IR frequencies (cm^-1^) of **[HL]**, **(1)** and **(2).**

compound	ν(N-H)	ν(C=N)	ν(C-O)	ν(Co-O)
HL	3421	1620	1161	-
Co(HL)_2_Cl_2_ **(1)**	3425	1619	1146	535
Co(HL)_2_Cl_2_ **(2)**	3425	1621	1147	534

HL:N-(2-hydroxy-1-naphthylidene)-2-methyl aniline
